# Protective efficacy of multivalent replication-abortive vaccine strains in horses against African horse sickness virus challenge

**DOI:** 10.1016/j.vaccine.2017.06.023

**Published:** 2017-07-24

**Authors:** Valeria Lulla, Andres Losada, Sylvie Lecollinet, Adeline Kerviel, Thomas Lilin, Corinne Sailleau, Cecile Beck, Stephan Zientara, Polly Roy

**Affiliations:** a Department of Pathogen Molecular Biology, Faculty of Infectious and Tropical Diseases, London School of Hygiene and Tropical Medicine, United Kingdom; b Université Paris-Est ANSES Alfort, UMR 1161 Virologie ANSES, INRA, ENVA, Maisons-Alfort, France

**Keywords:** AHSV, Orbivirus, Reverse genetics, Novel vaccine

## Abstract

African horse sickness virus (AHSV) is an orbivirus, a member of the *Reoviridae* family. Nine different serotypes have been described so far. AHSV is vectored by *Culicoides* spp. to equids, causing high mortality, particularly in horses, with considerable economic impacts. For development of a safe attenuated vaccine, we previously established an efficient reverse genetics (RG) system to generate Entry Competent Replication-Abortive (ECRA) virus strains, for all nine serotypes and demonstrated the vaccine potential of these strains in type I interferon receptor (IFNAR)-knockout mice. Here, we evaluated the protective efficacies of these ECRA viruses in AHSV natural hosts. One monoserotype (ECRA.A4) vaccine and one multivalent cocktail (ECRA.A1/4/6/8) vaccine were tested in ponies and subsequently challenged with a virulent AHSV4. In contrast to control animals, all vaccinated ponies were protected and did not develop severe clinical symptoms of AHS. Furthermore, the multivalent cocktail vaccinated ponies produced neutralizing antibodies against all serotypes present in the cocktail, and a foal born during the trial was healthy and had no viremia. These results validate the suitability of these ECRA strains as a new generation of vaccines for AHSV.

## Highlights

• AHSV is an emerging insect-borne virus.
• The mortality rate of infected horses is often up to 90%
• New generation of vaccines are made using reveres genetics.
• These vaccines are replication abortive but highly immunogenic.
• Both monovalent and multivalent vaccines are protective in ponies.

## 1 Introduction

African horse sickness (AHS) is an infectious arthropod-borne viral disease of equids, such as horses, donkeys, mules and zebras. In naïve horses, AHS virus (AHSV), a member of orbivirus genus, causes different forms of disease ranging from mild fever to an acute form, characterized by high fever, respiratory distress, lethargy and a mortality rate of over 90% [1]. AHSV is endemic in sub-Saharan Africa, although in the past, periodic outbreaks of AHSV have occurred in North African countries and southern Europe, such as Spain and Portugal [2]. Like closely related Orbiviruses such as bluetongue virus (BTV) and epizootic haemorrhagic disease virus (EHDV), AHSV is vectored by *Culicoides* midges, some species of which are present in Europe and the United states. In view of the recent emergence of BTV into Northern Europe, a region considered low risk for BTV introduction, the spread of AHSV outbreaks in Northern countries is a possibility. In order to control the disease in Africa, vaccination with a polyvalent live-attenuated vaccine (LAV) is currently used but it is considered to be unsafe due to the possibility of virulent revertants and the potential of reassortment between LAV and wild-type AHSV strains [3], [4].

AHSV is a non-enveloped virus containing a genome of 10 double-stranded RNA (dsRNA) segments (S1 to S10). Nine serotypes of AHSV (AHSV1 to AHSV9) have been described, whereas more than 27 BTV serotypes have been reported to date [5]. At sequence level, these viruses are highly diverse, however, AHSV is structurally closely related to BTV [6]. The double capsid viral particle is composed of seven proteins organized in concentric layers: an outer capsid, comprised of two major structural proteins VP2 and VP5 and an inner capsid, termed a core, consisting of a surface layer, made of VP7 and an inner layer, made of VP3. The inner layer encloses the replication complex consisting of three proteins, VP1, VP4, and VP6 and the genome of 10 dsRNA segments [7]. The design of safe AHSV vaccines, mainly based on the AHSV neutralization protein, VP2 protein, have been developed using various live vectors or as recombinant subunit vaccines but none have yet been commercialized [8], [9], [10]. Furthermore, since VP2 is serotype specific, AHSV vaccines for all nine serotypes are required in order to achieve protections against infection by all AHSV serotypes.

Reverse genetics (RG) is one of the most powerful tools to decipher viral replication processes and information gained by its use could be utilized for the development of novel and highly efficacious multivalent vaccines and a RG-based, live-attenuated AHSV4 vaccine candidate has shown promising results [11]. Our aim here was to produce replication-abortive viruses, which could not replicate in the vaccinated host and thus, significantly decrease the risk of viremia and potential reassortments with wild-type viruses. Indeed, development of such vaccine candidates were possible for BTV using a synthetic RNA-based RG system [12]. We demonstrated that while these vaccine strains were highly efficient to enter, but were unable to replicate in normal cells as genomic dsRNA molecules are not synthesised. These Entry Competent Replication Abortive (ECRA) virus strains (formally known as DISC) are deficient in VP6 (encoded by S9), and cannot complete even a single replication cycle. However, they still initiate the replication cycle and synthesize a single round of viral mRNAs following entry and express viral proteins in normal cells. Vaccine production of such strains is achieved by growth in a helper cell line that expresses VP6 *in trans* [13]. The protective abilities of such BTV strains were assessed in BTV-susceptible sheep demonstrating that strong protection against virulent virus challenge was afforded in the vaccinated animals. Subsequently, several vaccine trials in sheep and cattle were undertaken and the cumulative data confirmed the stability, protective efficacy and safety of these strains and their suitability as next generation vaccines for BTV [13], [14], [15].

With the aim of developing highly efficient candidate vaccines against all AHSV serotypes, particularly taking into account immunogenicity, safety and virus productivity aspects, we first established a highly efficient RG system for AHSV serotype 1, AHSV1 [16]. Subsequently, replication-abortive AHSV strains (ECRA.AHSV) of each of the nine serotypes were generated by introducing multiple stop codons in the coding region of segment S9, disrupting the ORF encoding VP6, an essential catalytic component of genome replication as well as the overlapping ORF of the NS4 protein [16]. Preliminary protective efficacy studies with these VP6-deficient virus strains in AHSV-sensitive mice lacking the type I interferon receptor (IFNAR), showed that vaccinated mice were protected against homologous virulent virus challenge, demonstrating the suitability of the ECRA-AHSV variants as a vaccine candidate. In this study, we sought to extend the vaccine protection trials to ponies, the natural host of AHSV. Two different vaccine regimes, a monoserotype (ECRA.A4) vaccine and a multivalent, cocktail vaccine of 4 different AHSV serotypes (ECRA.A1/4/6/8) were used in ponies followed by virulent virus challenge with one serotype. Results from clinical and immunological analyses demonstrated the protection efficacies of ECRA vaccines in ponies. Further, our data suggest that a cocktail of RG-based ECRA-AHSV strains may be suitable to be used as a multivalent vaccine in equids.

## 2 Methods

### 2.1 Cell lines and viruses

BSR cells (BHK-21 subclone) were maintained in Dulbecco modified Eagle medium (DMEM, Sigma) supplemented with 5% foetal bovine serum (FBS; Invitrogen). The stable cell line BSR-VP6 was generated by electroporation of AHSV1-VP6 expressing vector (pCAG-AHSV1 VP6), grown in DMEM supplemented with 5% FBS, and with 7.5 µg/ml of puromycin (Sigma) and tested by immunoblotting analysis [16], [17].

### 2.2 Recovery of replication-abortive AHSV variants

VP6-deficient AHSV1 (ECRA.A1) was generated as previously described [16]. Briefly, BSR cells stably expressing AHSV VP6 were transfected with 5 pCAG plasmids carrying the genes of VP1, VP3, VP4, VP6 and NS2, followed by transfection of 10 capped AHSV1 RNA transcripts: S1 to S8, S10, and S9multistop. To generate ECRA.AHSV strains for other serotypes, several segments were used to generate serotype-specific reassortants, by replacing equivalent segments in parental AHSV1 as described [16]. Each deficient virus was plaque-purified and titrated on BSR-VP6 cells.

For vaccination of ponies, the early passaged ECRA.AHSV stocks (1–2 passages in BSR-VP6 cells) were amplified in BSR-VP6 cells at an MOI 0.1 in antibiotic-free media and collected when complete cytopathic effect (CPE) was observed (48–72 h post infection). The resulting ECRA.AHSV stocks were supplemented with 10% trehalose (Sigma) and 1 ml aliquots were frozen in liquid nitrogen and stored at −80 °C until vaccination. The aliquots of frozen stocks were titrated on BSR-VP6 cells and tested for the absence of replication in wild type BSR cells for at least 3 blind passages by visualization of the absence of typical cytopathic effects and confirmation by RT-PCR. The mock-vaccine doses were prepared from an equivalent amount of BSR-VP6 cells via lysis by sonication.

### 2.3 Monoserotype and cocktail ECRA.AHSV vaccination in ponies

For vaccination, local breed ponies (poneys français de selle) were purchased from a farmer in Normandy, France, most of which were female and 2 geldings. Ponies were divided into two groups of 4 animals, A and B groups. Ponies were between 1 and 3 years old, except two (A4 and B4) that were older, over 10 years old. The weight of ponies were also varied between 72 kg and 164 kg. Ponies were subcutaneously inoculated with ECRA vaccine strains, group A with ECRA.A4 virus (1 × 10^7^ Plaque Forming Unit (PFU)/animal) and group B with a cocktail of ECRA.A1/4/6/8 (1 × 10^7^ PFU of each serotype/animal) at day 0. In addition, 2 animals were mock-vaccinated with uninfected cell lysates (Group C). A booster injection was given 21 days after the first vaccination. At day 36, two weeks after the booster vaccination, all animals were challenged intravenously with 2 × 10^6^ TCID_50_ of a virulent isolate of AHSV4 (Morocco 1990) (kindly provided by José Manuel Sánchez-Vizcaíno, Universidad Complutense de Madrid, Spain). Challenge virus stock was obtained after 5 passages on Vero cells of a spleen extract from an AHSV4 infected horse and subsequently passaged in *Culicoides* cells (KC) once. Whole-blood and serum samples of all animals were taken at regular intervals before and after vaccination and challenge. This study was performed in strict accordance with the French guidelines and recommendations on animal experimentation and welfare. The protocol was approved by the ANSES/ENVA/UPEC Animal Ethics Committee (Permit Number: 09/02/16-9, APAFiS 2016012115476384).

### 2.4 RNA extraction and RT-PCR

RNA was extracted from blood samples and from organs (spleen, lung and heart) homogenized in 10%w/v PBS with the QIAamp viral RNA kit and the Qiacube robot (QIAGEN). Genomic double-stranded RNA extracts were heat treated prior to addition to the RT-PCR reaction mix (addition of 10% DMSO and heating at 95 °C for 5 min). Real-time AHSV RT-PCR targeting the S1 segment was performed twice in three replicates according to established method [18], with modifications necessitated for the RT-PCR kit (AgPath-ID™ One-Step RT-PCR Kit (ThermoFisher Scientific) instead of SuperScript III/Platinum Taq One-Step qRT-PCR Kit) and to the cycling conditions in StepOne or AB7300 thermocycler: 45 °C for 10 min, 95 °C for 10 min followed by 45 cycles of 95 °C for 10 s, 55 °C for 30 s and 72 °C for 30 s. Cycle threshold (Ct) values were measured and values above a threshold of 40 were considered as negative.

### 2.5 Serology of monoserotype ECRA.A4 and cocktail ECRA.A1/4/6/8 trials

Serum samples were analysed by a commercially available competitive ELISA AHSV VP7 Antibody Test kit (ELISA Ingezim AHSV Compaq) according to manufacturer’s instructions. Serological status was defined as positive (≥50%), doubtful (>45% and <50%) or negative (≤45%). To detect neutralizing antibody response, standard serum neutralization (SN) assay was used as described previously [13] and titers were expressed as the reciprocal of the highest dilution of sera allowing complete neutralization of 100 AHSV PFU.

### 2.6 Clinical monitoring

Ponies were monitored on a daily basis during 8 days after vaccination and 3 weeks after challenge for development of AHSV clinical signs. General signs (behavioural modifications, hyperthermia, cardiac rate and breathing rhythm, sudation), as well as signs of oedema, abnormal bleeding (petechiae), dyspnoea, nasal discharge and conjunctivitis were recorded and scored according to the following criteria:

Behaviour: Normal – 0pt, Apathy – 1pt, Depression – 2pt, Prostration – 3pt
General parameters: rectal temperature (T) = Normal – 0pt, 39 °C ≤ T ≤ 40 °C – 1pt, T > 40 °C – 3pt; cardiac rate = Normal – 0pt, >50 – 2pt; breathing rhythm = Normal – 0pt, >25 – 2pt
Specific signs: Oedema – 1pt per location (eyelids, supraorbital fossa, lips, head, neck, trunk or disseminated), nasal discharge – 1pt per type of secretion (serous, mucous, purulent, haemorrhagic), petechial lesions – 1pt per location (conjunctivitis, oral cavity, skin), dyspnoea – 1pt, cough – 1pt, conjunctivitis – 1pt, abnormal sudation – 1pt, colic – 1pt.

## 3 Results

### 3.1 Preparation of ECRA vaccine strains of AHSV serotypes

**Fig. 1:**
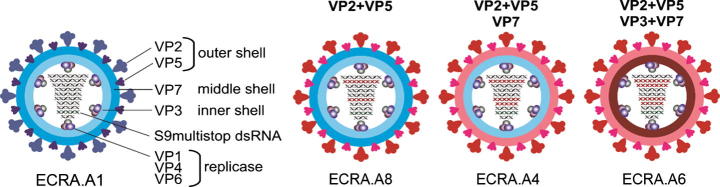
Vaccine Strains preparation. Schematic representation of the ECRA.AHSV vaccine strains obtained by reassortment. Vaccine strains ECRA.AHSV 4, 6 and 8 were obtained by reassortment of segments encoding the structural proteins (VP2, VP3, VP5, VP7) as indicated (modified from [1]).

By using the established BSR-VP6 complementary cell line, ECRA.AHSV1 could be recovered by RG through transfection of 5 viral protein expression plasmids followed by transfection of 10 capped T7 RNA transcripts, where S9 was replaced by the S9multistop segment disrupting the VP6 and NS4 ORFs [16]. After confirming that VP6 was efficiently complemented, we utilized the ECRA.AHSV1 strain as a backbone to produce deficient strains for all AHSV serotypes (2–9) by combining different segments. The exchange of 2 segments (S2 + S6) allowed the recovery of ECRA.A8 but 3 segments (S2 + S6 + S7) were required for the recovery of ECRA.A4 and 4 segments (S2 + S6 + S7 + S3) for the recovery of ECRA.A6 respectively (Fig. 1). Each vaccine strain was grown in BSR-VP6 complementary cells, titred on the same cells and stored in stabilised form [14] until the day of vaccination. For monoserotype vaccination 1 × 10^7^ PFU (ECRA.A4) were used per dose whereas the cocktail vaccination (ECRA.A1/4/6/8) contained 1 × 10^7^ PFU of each of the requisite ECRA-AHSV strains per dose. The particular cocktail mix of vaccine strains was selected based on the serological distances among these 4 serotypes.

### 3.2 ECRA vaccine strains do not replicate in ponies but trigger immune responses

To assess the protective efficacy of the ECRA.AHSV strains against AHSV4 infection in ponies, two groups (A & B) of 4 ponies were immunized twice with the 1 × 10^7^ PFU (ECRA.A4, group A) or a total of 4 × 10^7^ PFU for cocktail (ECRA.A1/4/6/8, group B). Uninfected, clarified cell lysates were inoculated to the control animals (group C). Due to restrictions on available animal numbers (10 ponies), only one cocktail of four serotypes was tested in this study. The primary vaccination was performed at day 0 (“prime”), and a repeated dose was inoculated for the booster vaccination at day 21 (“booster”).

**Fig. 2:**
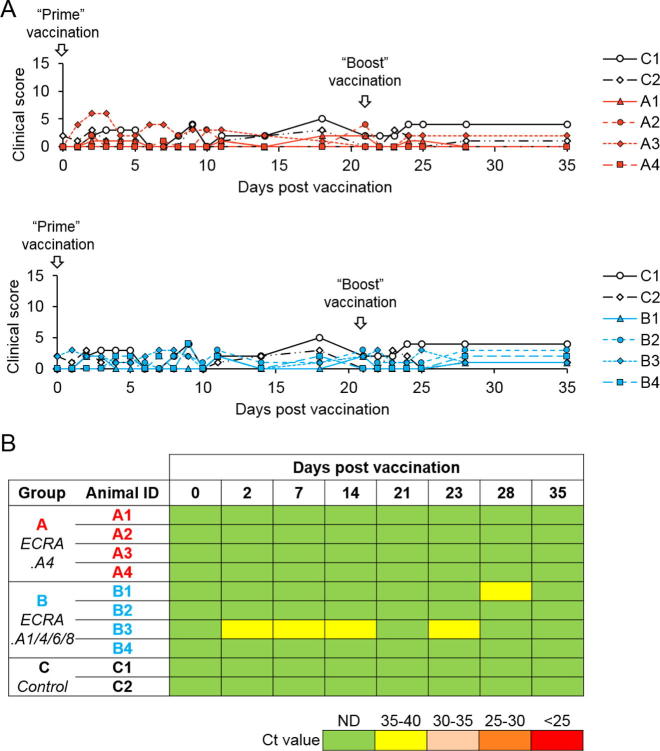
Clinical signs and viral replication in vaccinated animals. Animals were vaccinated twice at Day 0 and Day 21 (boost). (A) Clinical signs were scored based on body temperature, breathing rhythm, cardiac pulses, etc. Four animals were vaccinated with ECRA.A4 (group A; top panel), four with a cocktail of ECRA.A1/4/6/8 (group B; lower panel) and two animals were used as control (C1 & C2). (B) AHSV genomic RNA in serum was determined by RT-PCR and expressed as Ct values. Virus load is represented by a colour gradient from green (ND: not detected) to red (Ct less than 25). (For interpretation of the references to colour in this figure legend, the reader is referred to the web version of this article.)

Vaccinated ponies were routinely monitored from day 0 to day 35 but none showed any visible AHSV clinical reaction (Fig.2A). Similarly, the control non-vaccinated animals showed no reactions. Thus, the ECRA.AHSV vaccine strains were well tolerated and induced no adverse effect following immunization. Blood samples were collected periodically to monitor viral load and antibody production from day 0. Viral replication was monitored by RT-PCR of the blood samples of the animals, as previously described [18]. As shown in Fig.2B, from day 0 to day 35 viral RNA was either not detectable at all or present at a very low level in the vaccinated animals in both groups and were equivalent to the control animals. The data indicated the absence of viral replication after two doses of vaccination.

**Fig. 3:**
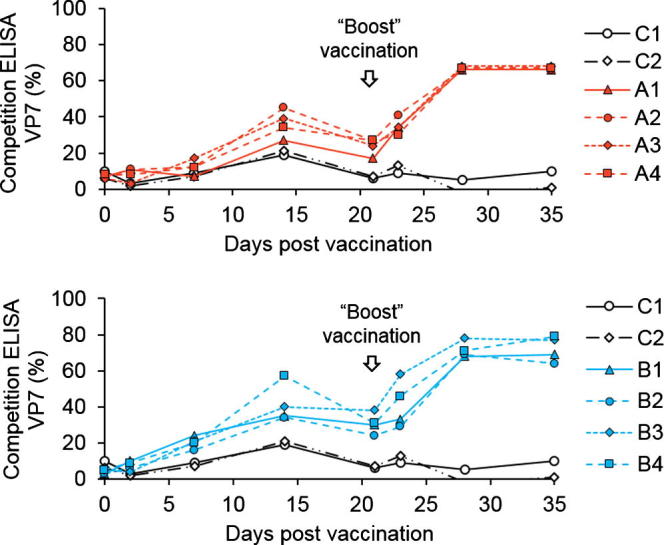
Seroconversion of animals after vaccination. The immune response of the vaccinated animals were monitored by VP7 group specific competitive ELISA. Animals in group A (top panel) and group B (bottom panel) were vaccinated twice with ECRA.A4 or ECRA.A1/4/6/8 respectively, 21 days apart and compared to group C (mock vaccinated animals).

Development of an antibody response to the ECRA.AHSV strains in ponies was initially monitored by a standard AHSV group specific VP7 antigen ELISA test. After the first vaccination, an immune response in the animals was evident, which was enhanced after a second vaccination (Fig. 3). All vaccinated animals of both groups A and B were seroconverted and a high level (up to 80%) of antibodies against VP7 could be detected at day 28 and day 35, i.e., at day 7 and 14 respectively after the second injection (Fig. 3). The sera from the control, non-vaccinated animals (C1 & C2) had no detectable VP7 antibody.

The immune response was further analysed by determining the neutralization antibody titers in vaccinated versus control animals (Table 1A). Serum samples collected at day 21 had very low, if any, neutralizing antibody (NA) titer against any serotypes (data not shown). However, sera collected at day 35 (two weeks after the booster vaccination and prior to challenge) of ECRA.A4 monovalent-vaccinated animals had NA titers ranging from 8 to 64 against AHSV4 (Table 1A). All animals in group B, vaccinated with the cocktail vaccine, had NAs against the 4 serotypes present in the vaccine cocktail, although the titers to each serotype varied (from 4 up to 64) from animal to animal (Table 1A), indicating that ECRA vaccine strains triggered neutralizing antibody responses in vaccinated animals.

**Table 1A:** Serum neutralization activity in ponies after vaccination. Neutralizing activity in sera was determined by SN assay at Day 35 (one day before challenge) against AHSV serotypes 1, 4, 5, 6 and 8 as indicated. Titers were expressed as the reciprocal of the highest dilution of sera allowing complete neutralization. Not determined (nd) and not detected (-) are indicated.

Group	Animal ID	Neutralizing antibody titers				
		Day 35 of vaccination				
		AHSV 1	AHSV 4	AHSV 5	AHSV 6	AHSV 8
A *ECRA.A4*	A1	*nd*	64	*–*	*nd*	*nd*
	A2	*nd*	32	*–*	*nd*	*nd*
	A3	*nd*	64	*–*	*nd*	*nd*
	A4	*nd*	8	*–*	*nd*	*nd*

B *ECRA .A1/4/6/8*	B1	16	16	*–*	8	8
	B2	8	16	*–*	4	8
	B3	16	64	*–*	32	16
	B4	4	8	*–*	8	4

C *Control*	C1	*–*	*–*	*–*	*–*	*–*
	C2	*–*	*–*	*–*	*–*	*–*

### 3.3 Protection against virulent virus challenge in vaccinated animals

To determine the protective efficacy of the vaccine strains, two weeks after the booster vaccination, control and vaccinated animals were challenged with 2 × 10^6^ TCID_50_ of virulent AHSV4, the most pathogenic virus of the serotypes used. To monitor virus load and neutralizing antibody production, blood samples were collected from each animal at regular intervals until 4 weeks after the challenge. One pony of the cocktail vaccinated group, B4, was found to be pregnant during the trial and was monitored for virus replication until the birth of the foal.

High titers of neutralizing antibodies against AHSV4 were detected at day 44 (8 days post challenge) in both group A and group B animals and some sera had NA titers against AHSV4 as high as 256 (Table 1B). The serum of pony A4, of group A, had an increased level of neutralizing antibody titer against AHSV4 by day 8 after challenge which increased to 256 at day 60 (24 days after challenge) (Table 1B). Neutralizing antibody titers up to 32 were also detected 8 days after challenge against AHSV1, AHSV6 and AHSV8 in certain ponies of group B. Twenty-four days after challenge (day 60 after vaccination) neutralizing antibodies against AHSV4 were generally higher after the challenge in all animals of both groups, presumably due to the memory response triggered by the challenge virus. The B group ponies that elicited neutralizing antibodies against AHSV1, AHSV6 and AHSV8 after vaccination also sustained neutralizing antibody titers up to 32 at day 60 (Table 1B), indicating the immune response of the vaccine strains in natural hosts, either when used individually or in a cocktail. No neutralizing antibodies were detectable in the two control animals (C1 and C2) (Table 1B).

**Table 1B:** Serum neutralization activity in ponies after virulent virus challenge. Neutralizing antibody titers of vaccinated animal sera were determined by SN assay at day 8 and day 24 (44 and 60 days post challenge) against serotypes AHSV 1, 4, 5, 6 and 8. Titers were expressed as the reciprocal of the highest dilution of sera allowing complete neutralization. Both C1 and C2 ponies were euthanized respectively at 11 days and 10 days’ post challenge due to severe AHS symptoms. Not determined (nd) and not detected (–) are indicated.

Group	Animal ID	Neutralizing antibody titers									
		8 days after challenge					24 days after challenge				
		AHSV 1	AHSV 4	AHSV 5	AHSV 6	AHSV 8	AHSV 1	AHSV 4	AHSV 5	AHSV 6	AHSV 8
A *ECRA.A4*	A1	*nd*	64	*nd*	*nd*	*nd*	*nd*	32	*nd*	*nd*	*nd*
	A2	*nd*	32	*nd*	*nd*	*nd*	*nd*	16	*nd*	*nd*	*nd*
	A3	*nd*	≥256	*nd*	*nd*	*nd*	*nd*	64	*nd*	*nd*	*nd*
	A4	*nd*	64	*nd*	*nd*	*nd*	*nd*	≥256	*nd*	*nd*	*nd*

B *ECRA .A1/4/6/8*	B1	16	32	–	4	8	8	16	–	4	8
	B2	–	64	–	–	8	4	32	–	–	4
	B3	4	≥256	4	16	32	4	128	–	16	32
	B4	–	≥256	4	–	8	–	32	–	–	–

C *Control*	C1	–	4	–	–	–	*nd*	*nd*	*nd*	*nd*	*nd*
	C2	–	–	–	–	–	*nd*	*nd*	*nd*	*nd*	*nd*

**Fig. 4:**
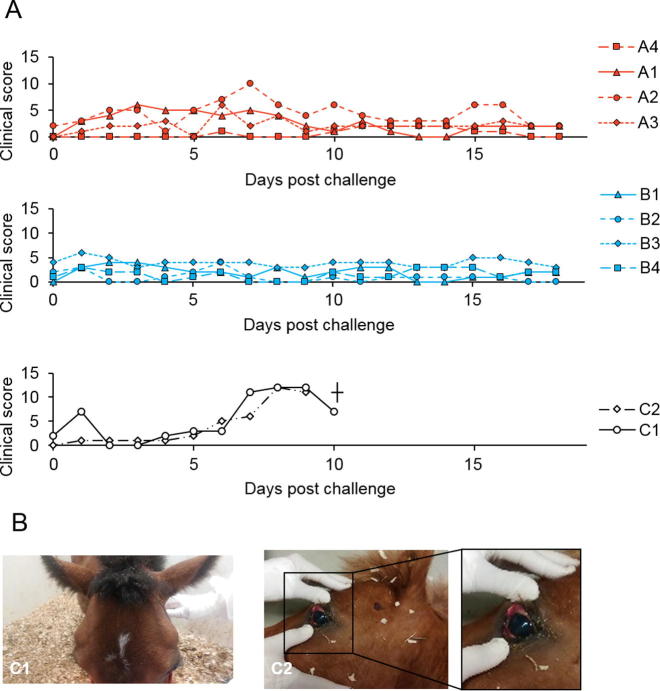
Clinical protection in vaccinated ponies after virulent virus challenge. Clinical signs were monitored from day 36 onwards. (A) Clinical signs were scored for vaccinated groups A and B (up to 18 days) and control group (up to 10 days) (upper, middle and lower panels, respectively). (B) Severe clinical signs after challenge in both control animals were evident as oedema of the eyelids and supraorbital fossae (left) and conjunctivitis (right).

None of the vaccinated animals in either group A or group B showed any significant clinical signs post challenge (Fig.4A top and middle panel respectively). One vaccinated animal, A2, showed mild circulatory (oedema of the eyelids, nasal discharge and moderate dyspnoea) and respiratory symptoms for two days only (6–8 days’ post challenge) (Fig.4A, top panel). This was probably due to partial protection afforded by lower neutralizing antibody level developed after vaccination (Table 1B). As expected both control animals started to show clinical signs shortly (6–7 days) after virulent virus challenge, with apathy, high hyperthermia and respiratory distress, oedema of the eyelids and supraorbital fossae as well as nasal discharge. These animals were euthanized at 10 days (C2) and 11 days (C1) post challenge respectively (Fig.4B). Necropsy performed on both control animals highlighted marked pulmonary oedema and pericardial and pleural effusions, which are consistent with AHSV infection.

**Fig. 5:**
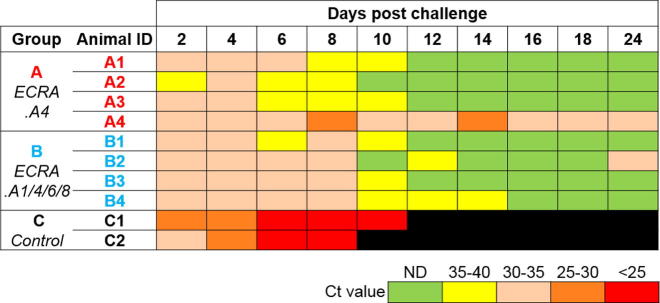
Absence of virus replication in vaccinated animals. Viremia in blood samples was determined by RT-PCR. Ct values are represented with a colour gradient from green (high) to red (low) corresponding low to high viral loads. Values above 40 were considered as not detected (ND). Control animals in group C (C1 & C2) were respectively euthanized at 11 and 10 days post challenge, due to severe AHS symptoms (black). (For interpretation of the references to colour in this figure legend, the reader is referred to the web version of this article.)

Both animals of the control group showed high viral loads (Ct values of 26.9 and 27.1), by RT-PCR, 4 days after challenge which increased until 8 days after challenge (Ct of 17.5 and 15.6) (Fig. 5), consistent with the severe clinical symptoms observed in these ponies (Fig.4A, bottom panel, and 4B). RT-PCR analysis of post mortem animals detected the presence of AHSV4 RNA in the hearts, spleens and livers of C1 and C2 ponies (Ct from 14.85 to 20.37, data not shown). In contrast, all vaccinated animals of group A and group B showed very low levels of viral RNA from 2 to 8 days after challenge with virulent virus strain (Ct from 31.4 to 37.2). For certain animals e.g. A4 and B4, RT-PCR signals consistent with a transient, low level viremia, were detected (Ct from 30.1 to 38.1, Fig. 5), 12 days after the challenge. However, no CPE was observed after 4 passages of the blood in Vero cells and no live virus was ever recovered, suggesting *de facto* protection from viremia despite the low RT-PCR signals (data not shown). During the course of the study animal B4 was found to be pregnant, however, the foal born subsequent to the challenge showed no viral RNA detected by RT-PCR. The foal was born on day 59 after the first vaccination and presumably after 345 days (±7 days) gestation period. It is noteworthy that virulent virus challenge was performed on day 322 of gestation and the first and second vaccine doses were inoculated on day 286 and day 307 respectively, of gestation. The new born foal had AHSV maternal antibodies detected at day 60 post prime vaccination and was completely healthy without any sign of AHS disease. The data indicated that the challenged virulent virus did not cross the placenta.

Taken altogether, these data demonstrate the safety of both ECRA.A4 and ECRA.A1/4/6/8 vaccination and the clinical protection afforded to vaccinated animals against a virulent AHSV4.

## 4 Discussion

Although RG systems for orbiviruses have been developed fairly recently, they have revolutionized not only the basic understanding of orbivirus replication but also the field of vaccine design. In particular, several RG-based vaccines have been developed for BTV and several of these candidate vaccines afforded protective immunity when tested in BTV susceptible animals (reviewed in [19]). However, RG-based AHSV vaccines have not yet been shown to be protective in horses. Our study here is the first report of a horse study of a RG-based AHSV vaccine and shows great potential to control AHS disease. This is a significant finding in the context of the live attenuated vaccines (LAVs) that are used in South Africa routinely as recent reports suggest these LAV vaccines pose considerable risks, due to their high reversion rates and reassortment of their genome segments with circulating strains, as well as their potential transmission by vectors [4]. In our current study we used our recently developed ECRA vaccine strains to assess their protective efficacy in ponies [16]. Prior to vaccination, we established that AHSV-ECRA strains behaved similarly to previously reported BTV DISC (ECRA) vaccine strains, consistent with both BTV and AHSV vaccines lacking segment S9, an essential viral gene [13]. The ECRA.AHSV vaccines were incapable of replicating in any cell line except a VP6-complementing cell line and they did not revert to a full cycle replication-capable virus upon passage in tissue culture [16]. However, in AHSV-susceptible normal cells, ECRA AHSV strains enter as efficiently as the wild-type viruses and release the transcriptionally active core into the cytoplasm. Subsequently ECRA core particles synthesize mRNA transcripts only once, which in turn are translated to viral proteins, except VP6 (and NS4), since S9 is defective. But *de novo* negative strands and genomic dsRNA molecules are not generated, furthermore no functional core is assembled. Such replication abortive ECRA.AHSV particles were shown to afford complete protection against homologous virus infection in type I interferon receptor (IFNAR)-knockout mice [16].

In the current study, we found that vaccination of ponies with ECRA strains induced no adverse effect, as anticipated, but that all animals seroconverted, particularly after a second vaccination. Importantly, all vaccine strains were shown to be incapable of replicating in animals, as expected from the absence of the essential VP6 protein. Animals in group B, which were vaccinated with the multivalent vaccine, had NAb titers against AHSV1, AHSV4, AHSV6 and AHSV8, the four ECRA vaccine strains present in the cocktail. These ECRA.AHSV strains used the ECRA.A1 backbone with other exchanged segments, notably segment-2 expressing the neutralization protein, VP2. The development of neutralizing antibody responses against each serotype included in the multivalent vaccine (ECRA.A1/4/6/8) suggested no significant interference in the immune response to each vaccine strain, and thus indicate that more complex multivalent vaccines may be possible [11].

The multivalent vaccine did not elicit NA production to heterologous AHSV serotypes, such as AHSV5, most likely due to phylogenetic distance of the target from the immunising cocktail [20]. Thus, our data suggests that vaccine strains of all serotypes may be needed in the cocktails in order to provide protective neutralizing antibodies against all AHSV serotypes.

The absence of ECRA.AHSV replication in vaccinated ponies was examined by RT-PCR with Ct values at either undetectable, or detectable only at very low level (range of 35–40), which gradually became undetectable. The detection of AHSV RNA at a low level, by a S1 primer was most likely due to residual genomes from the vaccination and the dose inoculated was 1 × 10^7^ particles, which corresponds to the 1 × 10^7^ copies of each dsRNA genomic segment. Such an amount of RNA can still be detected in vaccinated animals for a short duration without any replication in contrast to the rapidly increasing signal of genome replication observed in non-vaccinated challenged animals.

The immune responses elicited by monovalent ECRA.A4 or multivalent ECRA.A1/4/6/8 were sufficient to confer protection against virulent AHSV4 challenge in ponies, with the exception of one animal, A2, which developed transient low level clinical symptoms, but subsequently recovered. Interestingly, no significant increase of viral load in the bloodstream of this animal was detected. In another animal of the same group, A4, viral RNA was detected transiently after challenge; however, no disease was observed. In contrast to the vaccinated animals, the two control animals suffered severe AHS clinical symptoms including hyperthermia, respiratory distress, oedema of the eyelids and supraorbital fossae, pulmonary oedema and pericardial as well as pleural effusions.

Animal B4 was discovered to be pregnant during the study but nonetheless tolerated the multivalent ECRA.AHSV vaccine and gave birth to a healthy foal 25 days after challenge. The foal was free of any detectable virus and was seropositive for antibodies to AHSV, presumably maternal antibodies. Transplacental transmission of AHSV and related viruses has been described [21], [22], [23]. While traditional LAV for BTV and AHSV can block viremia and reduce transplacental transmission [24], [25], [26], the vaccines themselves are teratogenic. Although requiring confirmation with higher numbers, our study indicates that the replication-abortive vaccine strains maybe safe in pregnant ponies and maternal antibodies can be beneficially transferred to the foal.

It is important to note that the production of the ECRA.AHSV vaccine can be undertaken at BSL-2 conditions, since no infectious materials are involved at any stage. Such a process would dramatically decrease cost in comparison to the killed vaccine, which requires the generation of high titers of pathogens in high containment laboratories with subsequent inactivation and additional quality control steps. The ECRA.AHSV vaccine titer was as high as ∼1 × 10^8^ PFU/ml, and thus corresponds to ∼10 doses which were harvested from a culture of 1 × 10^7^ BSR-VP6 cells. Thus, such vaccines are not only cost efficient but likely to be manufactured rapidly as required.

During the last two decades, many attempts have been made to develop safe and efficacious AHSV vaccines. Such vaccines have the potential to be modified further for DIVA (Differentiate Infected from Vaccinated Animals) and could significantly reduce the possibility of recombination or reassortment with field strains and vector transmission. ECRA.AHSV vaccines are present in the vaccinated host only transiently as they fail to replicate yet provide a sufficient immune response to enable protection. Such vaccines have the safety profile of inactivated vaccines but the cost advantage and innate immunity triggering properties of attenuated vaccines. Taken together, replication abortive vaccine strains are excellent candidates for commercial development to control AHS.

## 5 Conclusion

We generated replication-abortive AHSV strains and assessed their protective efficacy in ponies. Vaccinated animals were protected against virulent virus challenge demonstrating the suitability of these deficient viruses as vaccines in animals. However, further studies are needed to understand the mechanisms of protective immunity, its longevity and to determine the minimum dose requirement in order to develop this vaccine platform for rapid responses to an outbreak with single or multiple AHSV serotypes.

## Funding

This work was funded by Biotechnology and Biological Sciences Research Council (BB/K015168/1).

## Conflicts of interests

The authors declare that they have no conflicts of interests.
